# Polydactyly and syndactyly in a Chinese family with Floating-Harbor syndrome: an expansion of the clinical phenotype

**DOI:** 10.3389/fgene.2026.1761836

**Published:** 2026-04-22

**Authors:** Junxiang Tang, Yanhong Cao, Daoqi Huang, Chaohong Wang, Xiaohua Jiang, Jiansheng Zhu

**Affiliations:** 1 Department of Medical Genetic, Anhui Women and Children’s Medical Center, Affiliated Maternity and Child Health Hospital of Anhui Medical University, Hefei, China; 2 Department of Obstetrics and Gynecology, The First Affiliated Hospital of USTC, Division of Life Sciences and Medicine, University of Science and Technology of China, Hefei, China; 3 Department of Reproductive, Anhui Women and Children’s Medical Center, Affiliated Maternity and Child Health Hospital of Anhui Medical University, Hefei, China

**Keywords:** Floating-Harbor syndrome, phenotype, polydactyly, SRCAP, syndactyly

## Abstract

Floating-Harbor syndrome (FLHS) is a rare neurodevelopmental and skeletal disorder caused by truncating variants in exons 33 and 34 of the *SRCAP* gene. It is characterized by distinctive facial features, delayed bone age, short stature, and moderate intellectual disability. While digital anomalies have been reported in approximately half of the more than 100 known cases, the phenotypic spectrum continues to expand. Here, we describe a family in which two individuals were identified with FLHS. Both the proband and her mother presented with typical manifestations, including classic facial dysmorphism, short stature, intellectual disability, brachydactyly, and clinodactyly. Moreover, the proband exhibited a novel combination of polydactyly and syndactyly affecting the right fifth and sixth toes, a feature previously unreported in FLHS. Additionally, she had complications including anemia, feeding difficulties, recurrent infections, epilepsy, and thrombosis. Whole-exome sequencing identified a heterozygous *SRCAP* c.7330C>T (p.Arg2444Ter) mutation in both affected individuals. The proband also harbored compound heterozygous mutations in *MMACHC* (c.609G>A/p.Trp203Ter and c.565C>T/p.Arg189Cys), potentially explaining some extra-skeletal symptoms. In summary, this study describes the first case of FLHS concurrently presenting with both polydactyly and syndactyly. Our work broadens the known phenotypic range of this rare syndrome.

## Introduction

**FIGURE 1:**
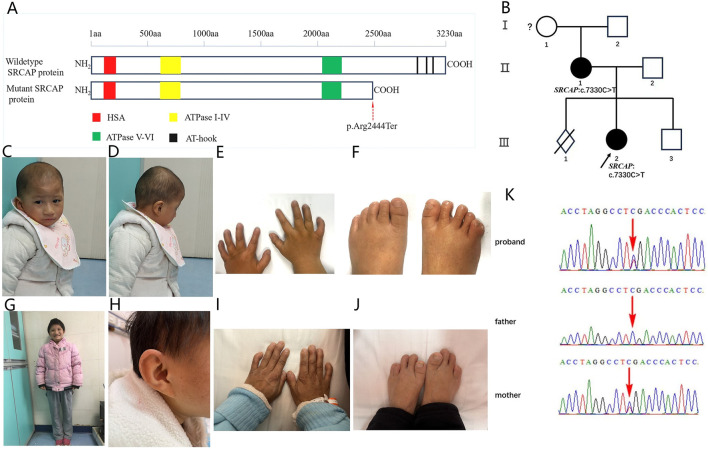
Clinical and molecular characteristics of the patients. **(A)** Schematic representation of wild-type and mutant SRCAP protein structures (GenBank: NP_006653.2). Protein domains are annotated with HAS(124–196) in red, ATPase I-IV (630–795) in yellow, ATPase V-VI (2,044–2,197) in green, and three AT-hooks (2,857–2,869; 2,936–2,948; 3,004–3,016) in black. The truncating mutation is indicated by red arrowheads. It introduces a premature termination codon, leading to a truncated protein that lacks the C-terminal region, including all three AT-hook domains. The amino acid scale is shown above the schematic. **(B)** Family pedigree of the proband and her mother. The black arrow indicates the proband. **(C,D)** Facial appearance of the proband showing a broad nasal tip, microretrognathia, and large ears. **(E,F)** Digit appearance of proband showing brachydactyly, broad fingertips, broad thumb, clinodactyly V finger, polydactyly, and syndactyly. **(G,H)** Appearance of the proband’s mother, showing short stature and distinctive facial features, including a broad nasal tip, microretrognathia, and large ears. **(I,J)** Digit appearance of the proband’s mother, showing brachydactyly, broad fingertips, broad thumb, and clinodactyly V finger. **(K)** Sanger sequencing revealed that the proband and her mother harbored a nonsense mutation c.7330C>T (p.Arg2444Ter) in the *SRCAP* gene. Red arrows indicate the variant base.

The *SRCAP* gene, located on chromosome 16p11.2, spans approximately 42 kilobases and comprises 34 exons. This gene encodes the core catalytic component of the multiprotein chromatin-remodeling *SRCAP* complex (Nagase et al., 1997). The SRCAP protein contains several conserved functional domains, including an N-terminal HSA domain, an ATPase domain divided into two sections containing conserved motifs I-IV and V-VI, respectively, and three C-terminal AT-hook motifs (Figure 1A) (White-Brown et al., 2023). It functions as an ATPase essential for incorporating the histone variant H2A.Z into nucleosomes, and it can also act as a transcriptional activator in Notch-mediated, CREB-mediated, and steroid receptor-mediated transcription processes (Johnston et al., 1999). Beyond its canonical role in chromatin remodeling, SRCAP also localizes to the mitotic apparatus, including centrosomes, the spindle, and the midbody, during cell cycle progression and is required for proper chromosome segregation and cytokinesis (Messina et al., 2021).

Variants in *SRCAP* were reported in a rare and severe skeletal syndrome, Floating-Harbor syndrome (FLHS) (OMIM # 136140). Although no consensus clinical diagnostic criteria for FLHS have been published, affected individuals have a markedly short stature with dysmorphic facial features. Language impairment, a high-pitched voice, mild to moderate intellectual disability, brachydactyly, broad fingertips, clinodactyly, and short thumbs are also commonly observed in patients (Nowaczyk et al., 1993). The frequent occurrence of brachydactyly and other digital anomalies in FLHS suggests that *SRCAP* plays a critical role in digital development. Polydactyly and syndactyly are among the most prevalent congenital digital abnormalities. Polydactyly results from disruptions in anteroposterior axis patterning during digital development, whereas syndactyly arises primarily from failure of programmed cell death (apoptosis) in the interdigital mesenchyme (Malik et al., 2010; Ahmed et al., 2017). These malformations can present as isolated, nonsyndromic entities with diverse inheritance patterns, or as components of more than 300 distinct syndromic disorders involving multiple organ systems (Mali and k, 2012). Remarkably, despite their prevalence across a wide range of conditions, the co-occurrence of polydactyly and syndactyly has not been documented in the digital phenotypic spectrum of FLHS. This observation prompted us to investigate whether this specific combination might represent an unreported manifestation of *SRCAP* mutations.

Here, we report two cases of FLHS in a Chinese family, in which the proband presented with both polydactyly and syndactyly, extending beyond the commonly recognized phenotypic spectrum of this disorder. To the best of our knowledge, this is the first report of genetically proven FLHS presenting with both polydactyly and syndactyly.

## Materials and methods

### Patients

The study evaluated a family with two members suffering from FLHS (Figure 1B).

### Whole-exome sequencing

The experimental procedure for trio-WES and the bioinformatics analysis has been described previously (Li et al., 2024). Briefly, genomic DNA was extracted from the patients’ peripheral blood and fetal uncultured amniotic fluid using the QIAamp DNA Blood Mini Kit according to the manufacturer’s instructions. A DNA library was prepared using Illumina protocols and sequenced on the NovaSeq 6,000 platform (Illumina, United States) in accordance with the manufacturer’s instructions. All exon regions and 20 bp of exon-flanking intron regions were captured for sequencing.

Quality control of the WES data was performed with fastq. Mapping (BWA) and variant calling (GATK, SAMtools, and FreeBayes) were streamlined using the SeqMule pipeline with the GRCh37 genome. Genetic variants were annotated by ANNOVAR. The mutations were classified according to the standards and guidelines of the American College of Medical Genetics and Genomics (ACMG/AMP 2015 guideline).

### Sanger sequencing verification of the *SRCAP* gene

PCR primers were designed to amplify and sequence the *SRCAP* gene (GenBank accession No. NM_006662.3) using Primer3 software (http://primer3.ut.ee/) and were synthesized by BGI (Shanghai, China). Primers designed for exon 33 were forward: 5′-ACC​CTG​TTG​TTG​TAG​GAG​CAA-3′ and reverse: 5′-CTC​TGG​AGA​TGC​CAA​TGC​CT-3′. The targeted exons and exon–intron boundaries were amplified via polymerase chain reaction. Sanger sequencing was performed with both sets of forward and backward primers using an ABI3730XL sequencer (Applied Biosystems, Foster City, CA, United States). Data were analyzed using Mutation Surveyor DNA Variant Analysis Software (SoftGenetics, LLC.).

## Results

### Patient description

The proband is an 8-year-old female. She was born at 35 weeks via natural childbirth due to premature birth with a birth weight of 1800 g (−2.29 SD)and a length of 40 cm (−3.6 SD). Notable prenatal findings at 23 weeks included a right subclavian vein variation, placental hemorrhage, a nuchal cord, and abnormally tight umbilical cord spirals. At birth, she had Apgar scores of 6 and 10 at 1 min and 5 min, respectively, leading to a diagnosis of neonatal asphyxia. She also presented with neonatal pneumonia, coagulation defects, anemia, a cleft palate, polydactyly/syndactyly of the right little toe, and hyperbilirubinemia. At the age of 1 year, she underwent surgery for congenital megacolon. She was later hospitalized for severe malnutrition, as well as femoral venous thrombosis and diarrhea, and persistent epileptic seizures with infection at 18 months.

She was found to have a developmental delay at age 3 years. Her height was 80 cm (−3.97 SD), and her weight was 10 kg (−3.73 SD). Physical examination revealed distinctive craniofacial features, including a short mandible, low-set ears, a large nasal tip, and sparse eyebrows (Figures 1C,D). Additionally, the patient had short fingers bilaterally, with clinodactyly of the fifth fingers. A combined syndactyly and polydactyly was noted on the right little toe (Figures 1E,F). Neuropsychological test results based on the Wechsler Preschool and the Primacy Scale of Intelligence revealed moderate intellectual disability. In terms of language, she could produce simple words and combine them into short phrases, but could not form complete sentences. Regarding motor skills, she could run slowly but was unable to run rapidly or perform consecutive jumps. Ultrasonography revealed bilateral renal stones.

A comparison between the proband and her mother revealed a less severe manifestation of the condition in the mother. The mother, aged 29, stands at 135 cm (−4.0 SD) and weighs 30 kg (−2.94 SD). Physical examination revealed a pointed mandible, triangular face, sparse eyebrows, large nasal tip, clinodactyly of the little fingers on both hands, and short fourth and fifth toes on both feet without polydactyly/syndactyly (Figures 1G–J). The mother also exhibits a speech developmental disorder. The mother had a history of one miscarriage prior to the delivery of the proband, who is her first live-born child.

When the proband was 3 years old, her mother conceived again. Prenatal genetic analysis was performed on amniotic fluid obtained by amniocentesis. Her mother delivered a male infant at term. The infant has shown no signs of developmental abnormalities to date. The proband’s father was highly myopic but was otherwise healthy. According to family reports, the maternal grandmother exhibited phenotypic similarities to the mother, including craniofacial dysmorphism and short stature, but could not be evaluated. The proband’s parents denied consanguinity.

### Identification of *SRCAP* variant

The proband and her mother were clinically diagnosed with a skeletal-neurological syndrome and were therefore subjected to trio-WES. After removal of common variants with allele frequencies >1% in the gnomAD database (http://gnomad.broad.institute.org/) and benign variants predicted by various *in silico* tools, such as PolyPhen-2, MutationTaster, and MaxEntScan, clinical features of developmental delay, intellectual disability, and short stature served as filtering indices to analyze the candidate variants. WES revealed that the proband harbored a heterozygous nonsense mutation (NM_006662.3: c.7330C>T) in *SRCAP*. The nonsense variant was inherited from her mother, while both alleles in her father were wild-type, as confirmed by Sanger sequencing (Figure 1K). Subsequently, we also performed WES on the amniotic fluid from the proband’s mother, which showed that the fetus (the proband’s younger brother) did not carry the nonsense mutation in *SRCAP*.

### Identification of *MMACHC* variants

WES revealed that the proband harbored a heterozygous missense variant (NM_015506.3: c.565C>T) and a heterozygous nonsense variant (NM_015506.3: c.609G>A) in the *MMACHC* gene. The missense variant was inherited from her mother, while the nonsense variant was inherited from her father, indicating that the proband harbored a compound heterozygous *MMACHC* variant. WES performed on the amniotic fluid showed that the fetus was heterozygous only for the nonsense variant in *MMACHC* (NM_015506.3: c.609G>A), a finding consistent with a carrier status.

### Pathogenicity analysis of the *SRCAP* variant

The c.7330C>T(p.Arg2444Ter) variant located at exon 34 of *SRCAP* was not present in the gnomAD database. It has been previously reported in patients with FLHS (Zhang et al., 2019). According to the ACMG/AMP 2015 guideline for clinical interpretation of genetic variants (Richards et al., 2015), the c.7330C>T variant was classified as pathogenic (PVS1_Strong, PS4, PS2_VeryStrong, PM2_Supporting).

### Pathogenicity analysis of the *MMACHC* variants

The c.609G>A (p.Trp203Ter) variant was located in exon 4 of the *MMACHC* gene and had an extremely low (0.0044%) allelic frequency in the gnomAD database. It has been previously reported in patients with methylmalonic acidemia (He et al., 2020). According to the clinical interpretation of genetic variants by the ACMG/AMP 2015 guideline, the c.609G>A (p.Trp203Ter) variant was classified as pathogenic (PM3_VreyStrong, PVS1_Strong, PM2_Supporting). The c.565C>T (p.Arg189Cys) variant was also located at exon 4 of the *MMACHC* gene with an extremely low (0.0012%) allelic frequency in the gnomAD database. It has also been previously reported in patients with methylmalonic acidemia (Wang et al., 2019). We classified this mutation as likely pathogenic, with arguments such as PM3_Strong, PM5, PM2_Supporting, and PP3.

## Discussion

FLHS is a genetic disorder resulting from truncating variants in exons 33 and 34 of the *SRCAP* gene. FLHS is characterized by typical craniofacial features, low birth weight, normal head circumference, short stature, bone age delay that normalizes between 6 years and 12 years of age, skeletal anomalies including brachydactyly, broad fingertips, clinodactyly, short thumbs, prominent joints, and clavicular abnormalities (Nowaczyk et al., 1993). As all identified mutations in FLHS patients are heterozygous *de novo* events that predictably truncate the *SRCAP* protein by removing its C-terminal DNA-binding motif, a dominant-negative mechanism, rather than haploinsufficiency, is postulated to be the underlying disease cause (Neu-Yilik et al., 2011). The vast majority of reported mutations in FLHS cluster in exon 34, with a minority found in exon 33. Interestingly, truncations outside this critical region (exons 33–34) trigger nonsense-mediated decay (NMD), potentially resulting in a distinct allelic variant and consequently different phenotypic effects such as developmental delay, hypotonia, musculoskeletal defects, and behavioral abnormalities (Rots et al., 2021). In Europe and North America, the predominant mutation is c.7330C>T (p.Arg2444Ter), which is identified in approximately half of all FLHS patients. The second most prevalent mutation, c.7303C>T (p.Arg2435Ter), is found in about one-quarter of the patient cohort (Seifert et al., 2014; Nikkel et al., 2013). In a Chinese patient cohort, however, the frequencies of these two variants showed no significant difference (5/12 vs. 4/12) (Zhang et al., 2019).

In this study, the nonsense mutation c.7330C>T (p.Arg2444Ter) was found in the proband and her mother. This mutation is recurrent in this family. The *SRCAP* c.7330C>T (p.Arg2444Ter) mutation introduces a premature termination codon and is predicted to result in a truncated protein that lacks AT-hooks by escaping NMD (Figure 1A). The AT-hook domain, in addition to being essential for direct DNA binding by epigenetic regulators, can also function as a nuclear localization signal. The truncated SRCAP protein competes with the wild-type protein for complex formation, thereby mislocalizing the entire *SRCAP* complex, which is an effect that likely contributes to the pathogenesis of FLHS (Rots et al., 2021; Messina et al., 2016; Hood et al., 2012).

In this study, the proband and her mother shared key diagnostic features, such as short stature, brachydactyly, clinodactyly, a triangular face, and a prominent nose, underscoring the familial transmission of the phenotype. Notably, phenotypic manifestations of FLHS vary across ethnicities. In the study by Zhang et al., delayed bone age was a consistent feature in all Chinese patients (12/12), whereas it was present in only approximately half of Western patients (38/68). Conversely, a low-hanging columella was observed in all Western patients (70/70) but in only approximately half of the Chinese cohort (7/12) (Zhang et al., 2019). The proband shared her mother’s characteristic facial features. However, the proband presented with polydactyly and syndactyly, whereas the mother’s phenotype did not include these digital anomalies. The occurrence of syndactyly in FLHS is exceedingly rare, with only isolated cases of syndactyly documented in the literature (Zhang et al., 2019; Seifert et al., 2014; Nikkel et al., 2013; Le Goff et al., 2013; Kehrer et al., 2014; Gerundino et al., 2014; Amita et al., 2016; Budisteanu et al., 2018; Ko et al., 2020; Son et al., 2020; Ercoskun and Yuce-Kahraman, 2021; Bo et al., 2021; Turkunova et al., 2022; Yang et al., 2023; He et al., 2025; Xiao et al., 2025). Moreover, there have been no documented clinical reports of patients with this disorder exhibiting both polydactyly and syndactyly concurrently (Table 1).

**TABLE 1:** Digit phenotypic comparison of our patients with reported patients.

Patients	No. of patients	Broad thumbs	Brachydactyly	Broad fingertips	Clinodactyly V finger	Syndactyly	Polydactyly	Middle phalange dysplasia
This study	2	2/2	2/2	2/2	2/2	1/2	1/2	-
Reported by Zhang et al. (reported/observed) (Zhang et al., 2019)	12	6/12	8/12	4/12	4/12	-	-	NR
Reported by Seifert et al. (reported/observed) (Seifert et al., 2014)	5	1/5	3/5	2/5	3/5	-	-	NR
Reported by Nikkel et al. (reported/observed) (Nikkel et al., 2013)	17	10/17	6/17	2/17	3/17	-	-	NR
Reported by Goff et al. (reported/observed) (Le Goff et al., 2013)	9^a^	-	1/9	-	6/9	3/9	-	NR
Reported by Kehrer et al. (reported/observed) (Kehrer et al., 2014)	1	-	-	-	+	-	-	NR
Reported by Gerundino et al. (reported/observed) (Gerundino et al., 2014)	1	-	-	-	+	-	-	NR
Reported by Amita et al. (reported/observed) (Amita et al., 2016)	1	-	+	-	-	-	-	NR
Reported by Budisteanu et al. (reported/observed) (Budisteanu et al., 2018)	1	-	+	+	-	-	-	NR
Reported by Jaemin et al. (reported/observed) (Ko et al., 2020)	1	-	+	-	-	-	-	NR
Reported by Son et al. (reported/observed) (Son et al., 2020)	1	-	-	+	+	-	-	+
Reported by Ercoskun et al. (reported/observed) (Ercoskun and Yuce-Kahraman, 2021)	1	-	-	+	+	-	-	NR
Reported by Bo et al. (reported/observed) (Bo et al., 2021)	1	-	+	-	+	-	-	+
Reported by Turkunova et al. (reported/observed) (Turkunova et al., 2022)	1	-	+	-	+	-	-	NR
Reported by Yang et al. (reported/observed) (Yang et al., 2023)	1	-	+	-	-	-	-	NR
Reported by He et al. (reported/observed) (He et al., 2025)	1	-	+	-	+	-	-	+
Reported by Xiao et al. (reported/observed) (Xiao et al., 2025)	1	-	-	+	-	-	-	NR

^a^ No mutations in the *SRCAP* gene were detected in three patients. NR: not reported. +: present. -: absent.

Polydactyly and syndactyly are among the most prevalent congenital digit anomalies. These malformations can occur as isolated traits or as components of numerous syndromic disorders (Mali and k, 2012). In this case study, the proband exhibited postaxial polydactyly/syndactyly of the fifth and sixth toes of the right foot, whereas her mother only presented with brachydactyly affecting both her hands and feet. However, our examination of the proband’s genetic sequencing data did not reveal any pathogenic variants in well-known polydactyly-associated genes, such as *HOXD13, FBLN1, GJA1,* and *BHLHA9* (Deng and Tan, 2015; Zaib et al., 2022).

Of note, the proband was found to harbor compound heterozygous variants in the *MMACHC* gene. Mutations in the *MMACHC* gene cause the cobalamin C defect (OMIM # 277400), which is the most common type of cobalamin metabolism disorder and is inherited in an autosomal recessive manner (Morel et al., 2006). This disorder impairs the conversion of cobalamin (vitamin B12) into its two active forms: adenosylcobalamin and methylcobalamin. These forms are essential cofactors for the enzymes that convert methylmalonyl-CoA to succinyl-CoA in the mitochondria and homocysteine to methionine in the cytosol, respectively. Consequently, this deficiency leads to the accumulation of homocysteine (HCY) and methylmalonic acid (MMA), along with normal or decreased levels of methionine (Ding et al., 2023). The most common manifestations of the disease include epilepsy, growth retardation, dystrophy, anemia, metabolic abnormalities, thrombocytopenia, microcephaly, dementia, and ophthalmic abnormalities (Rosenblatt et al., 1997). The incidence of the cblC defect ranges from 1:3,220 to 1:21,488 in China (Zhou et al., 2018).

To date, more than a hundred *MMACHC* gene mutations have been deposited in the human gene mutation database (HGMD, www.hgmd.cf.ac.uk/ac/index.php), most of which are missense/nonsense mutations, followed by small deletions (Hao et al., 2025; Lu et al., 2025). The most common *MMACHC* mutations in Chinese patients were c.80A>G (p.Gln27Arg), c.609G>A (p.Trp203Ter), c.482G>A (p.Arg161Gln), c.394C>T (p.Arg132Ter), and c.658_660del (p.Lys220del) (Wang et al., 2020).

The proband was identified as compound heterozygous for two mutations, c.609G>A (p.Trp203Ter) and c.565C>T (p.Arg189Cys), which were maternally and paternally inherited, respectively. Notably, her phenotype was more severe than that of her mother, incorporating additional manifestations such as anemia, failure to thrive, feeding difficulties, recurrent infections, epileptic seizures, and venous thrombosis. These clinical features raised a strong suspicion of an inborn error of metabolism, corroborated by subsequent metabolic profiling that revealed marked abnormalities in propionylcarnitine (C3), acetylcarnitine (C2), and methionine, C3/C2. Following initiation of therapy with vitamin B12, significant improvement was observed in multiple laboratory parameters (Table 2).

**TABLE 2:** The level of specific metabolites detected in the proband after 5 days of treatment.

Specific metabolites	Before treatment	After 5 days of treatment	Normal range
Methionine	6.89	8.37	8.50–45 μmol/L
Propionylcarnitine (C3)	7.52	3.31	0.40–5.00 μmol/L
Acetylcarnitine (C2)	7.49	8.88	5.0–55.00 μmol/L
C3/C2	1.00	0.37	0.03–0.20
Urinary methylmalonic	51.40	7.10	0–4.00 mg/dL
Homocysteine	62.6	48.1	3.0–20.0 μmol/L
Blood ammonia	128.5	52.8	18.0–70 μmol/L
Blood lactic acid	3.90	1.1	0.5–2.0 mmol/L

Collectively, these findings suggest that in addition to FLHS, the proband also has an additional diagnosis of cobalamin C defect caused by compound heterozygous mutations in the *MMACHC* gene. We propose that the proband’s polydactyly and syndactyly are attributable to the *SRCAP* mutation, expanding the phenotypic spectrum of FLHS, rather than to the *MMACHC*-related disorder. This interpretation is supported by clinical observations and mechanistic considerations. First, syndactyly has been previously documented in association with *SRCAP* mutations. Le Goff et al. reported three FLHS patients with syndactyly, among whom two harbored distinct pathogenic *SRCAP* variants, specifically c.7330C>T and c.7863dup (Table 1) (Le Goff et al., 2013).

The documentation of syndactyly in individuals with confirmed *SRCAP* mutations supports its inclusion in the FLHS phenotypic spectrum. Furthermore, the underlying pathomechanisms are fundamentally distinct. *MMACHC*-related disorders result from metabolic dysfunction affecting cobalamin metabolism (Wang et al., 2019). For our patient, however, the polydactyly and syndactyly may be explained by the role of SRCAP in cell division. In addition to its canonical function in chromatin remodeling, SRCAP localizes to the mitotic apparatus and is required for proper cytokinesis. Depletion of SRCAP in human cells leads to a range of mitotic and cytokinetic defects, such as chromosome misalignment, multipolar spindles, and the formation of multinucleated cells (Messina et al., 2021).

These findings offer a potential explanation for the developmental abnormalities observed in FLHS patients. Although the study by Messina et al. did not investigate digital development directly, we hypothesize that the cytokinesis defects caused by *SRCAP* mutations could extend to digital formation during embryonic development. Precise control of cell division is critical for the sculpting and separation of digits. Disruptions to this process can lead to an excess of cells, potentially contributing to polydactyly, or a deficit due to impaired interdigital cell death, which may result in syndactyly (Deng and Tan, 2015). The midbody, in particular, plays a critical role in the final separation of daughter cells. Messina et al. showed that SRCAP localizes to the midbody and is required for the recruitment of key cytokinesis regulators (Messina et al., 2021). Dysfunction of this recruitment mechanism could lead to failed abscission during digital development, contributing to soft tissue syndactyly or other toe abnormalities (Chopra et al., 2013; Cassim et al., 2022).

This hypothesis is further supported by the conserved role of SRCAP orthologs in *Drosophila*, in which DOM-A depletion similarly disrupts cell division, underscoring the fundamental importance of this gene in developmental processes across species (Messina et al., 2021). Thus, we propose that the combination of polydactyly and syndactyly may be previously unrecognized features of FLHS. In the future, systematic *in vitro* experiments to assess the effects of this nonsense mutation on the function of the *SRCAP* protein can help us better understand the phenotypic differences among patients.

In summary, we identified a Chinese FLHS pedigree in which the proband presents with a previously unreported digenic mutation involving both *SRCAP* and *MMACHC*. Moreover, the patient is the first reported case exhibiting polydactyly-syndactyly complex, an unprecedented feature that highlights the extensive phenotypic diversity of FLHS.

## Data Availability

The original contributions presented in the study are publicly available. This data can be found in the NCBI repository at https://www.ncbi.nlm.nih.gov/sra/PRJNA1442885, with the accession number PRJNA1442885.
